# Glucose variability is a marker for COVID-19 severity and mortality

**DOI:** 10.20945/2359-3997000000527

**Published:** 2022-10-11

**Authors:** Ran Abuhasira, Alon Grossman

**Affiliations:** 1 Tel Aviv University Sackler Faculty of Medicine Tel Aviv Israel Sackler Faculty of Medicine, Tel Aviv University, Tel Aviv, Israel; 2 Rabin Medical Center Department of Medicine B Petah Tikva Israel Department of Medicine B, Rabin Medical Center, Beilinson Campus, Petah Tikva, Israel

**Keywords:** Glucose variability, COVID-19, hyperglycemia hospital management

## Abstract

**Objective:**

We aimed to investigate the association between glucose coefficient of variation (CV) and mortality and disease severity in hospitalized patients with coronavirus disease-19 (COVID-19).

**Subjects and methods:**

Retrospective cohort study in a tertiary center of patients with COVID-19 admitted to designated departments between March 11^th^, 2020, and November 2^nd^, 2020. We divided patients based on quartiles of glucose CV after stratification to those with and without diabetes mellitus (DM). Main outcomes were length of stay and in-hospital mortality.

**Results:**

The cohort included 565 patients with a mean age of 67.71 ± 15.45 years, and 62.3% were male. Of the entire cohort, 44.4% had DM. The median glucose CV was 32.8% and 20.5% in patients with and without DM, respectively. In patients with DM, higher glucose CV was associated with a longer hospitalization in the unadjusted model (OR = 2.7, 95% CI [1.3,5.6] for Q4), and when adjusted for age, sex, comorbidities, and laboratory markers, this association was no longer statistically significant (OR = 1.3, 95% CI [0.4,4.5] for Q4). In patients with and without DM, higher glucose CV was associated with higher rates of in-hospital mortality in the unadjusted model, but adjustment for comorbidities and laboratory markers eliminated the association (OR = 0.5, 95% CI [0.1,3.4] for Q4 in patients with DM).

**Conclusion:**

Higher glucose CV was associated with increased in-hospital mortality and length of stay, but this association disappeared when the adjustment included laboratory result data. Glucose CV can serve as a simple and cheap marker for mortality and severity of disease in patients with COVID-19.

## INTRODUCTION

Coronavirus disease 2019 (COVID-19) was an emerging pandemic in 2020 caused by a novel coronavirus named SARS-CoV-2 (1,2). Diabetes confers a significant additional risk for COVID-19 patients (3–5). Additionally, dexamethasone, which can cause drug-induced hyperglycemia, has become the standard of care for patients with severe COVID-19 (6).

Glucose variability is the glycemic oscillation between time points: same-day, between-days, or within the same hospitalization (7,8). Several studies in recent years have shown that high glucose variability during hospitalization, measured as coefficient of variation (CV) and standard deviation (SD), was associated with increased mortality and length of hospital stay for non-critically ill and critically ill patients with sepsis and other patients with acute infections (9–13). It was also hypothesized that increased glucose variability plays a role in the more severe forms of influenza H1N1 (14). In a recent review, Monnier and cols. explored the consequences of short- and long-term glucose variability on cardiovascular outcomes (15).

Two major risk factors for severe COVID-19 disease and its complications are hypertension and diabetes (16–18). Initially, it was also postulated that anti-hypertensive medications would affect the outcome (19), but this was later refuted (20). The mechanisms underlying the risk of diabetes for severe COVID-19 are poorly understood. One of the suggested mechanisms is hyperglycemia increasing expressions of angiotensin-converting enzyme 2 (ACE2) protein and transmembrane protease serine 2 (TMPRSS2), both of which are required for viral entry into human cells (21–23). Another contributing factor is high glycemic variability, and the American Diabetes Association (ADA) issued specific recommendations for managing hyperglycemia in these patients (24). Zhu and cols. found that well-controlled blood glucose was associated with significantly lower mortality rates in hospitalized patients with COVID-19 in China (25). Two additional studies on hospitalized patients with COVID-19 showed that elevated fasting blood glucose on admission is associated with increased mortality (26,27). Whether high glucose variability is a risk factor for mortality and longer hospitalizations in COVID-19 patients with and without diabetes is unknown. The aim of this study was to investigate the association among glucose CV, in-hospital mortality, and length of stay for COVID-19 patients.

## SUBJECTS AND METHODS

### Ethics

The Rabin Medical Center institutional review board (IRB) Committee approved this study (confirmation number 0787-20). We conducted all clinical investigations according to the principles expressed in the Declaration of Helsinki. The IRB approval exempted the study from informed consent because the data collection's retrospective nature maintained subject confidentiality. We anonymized and de-identified patient records prior to analyses.

### Study design and population

We conducted a retrospective cohort study at a large, 1,300-bed university-affiliated tertiary medical center. The medical center had three COVID-19-designated departments and one COVID-19 intensive care unit (ICU). We included all patients 18 or older admitted with a positive SARS-CoV-2 PCR test between March 11^th^, 2020, and November 2^nd^, 2020. We excluded patients with fewer than three glucose measurements during their hospitalization, admission shorter than 24 hours, those still hospitalized on the day of data extraction, and those who were not admitted to a designated COVID-19 department from the analysis.

The standard of care for patients with diabetes in our institution is holding oral anti-diabetic drugs for all hospitalized patients and initiating insulin therapy with a basal bolus protocol, according to the ADA recommendations (28).

### Definitions and data sources

We extracted all data from the hospital's computerized medical record. Diabetes mellitus (DM) was defined by International Classification of Diseases, 9th revision (ICD-9) codes or by the use of glucose lowering drugs during the admission.

Confirmed SARS-CoV-2 was defined as a positive respiratory sample for RT-PCR for SARS-CoV-2, using commercial RT-PCR methods (either Seegene Allplex™ 2019-nCoV Assay (Seegene, Seoul, South Korea) or GeneXpert).

Glucose CV is determined by the ratio of glucose standard deviation to glucose mean value of each patient (7). We used all glucose values since admission that were documented in the medical record, whether capillary or venous, some of them being fasting measurements and others random glucose measurements. We divided glucose CVs of the entire cohort into quartiles based on the presence or absence of DM.

The study's endpoints were in-hospital mortality and hospital length of stay.

### Statistical analysis

We present the results as mean ± SD for continuous variables, medians and interquartile ranges for ordinal variables, and percentages for categorical data. We assessed associations among glucose CV, in-hospital mortality, and length of stay (length of stay over 7 days), adjusted for age, sex, comorbidities, and laboratory test results, with multivariable logistic regression. We also used multivariable logistic regression to create a prediction model for in-hospital mortality using age, sex, comorbidities, and laboratory test results but not glucose CV. We also assessed the same variables conducting Cox multivariable analysis. We then used the locally weighted scatterplot-smoothing (LOWESS) curve to assess a potential non-linear relationship of glucose CV and in-hospital mortality. We conducted statistical analyses using SPSS version 25.0 (IBM, Chicago, USA).

## RESULTS

### Study Population

**Figure 1:**
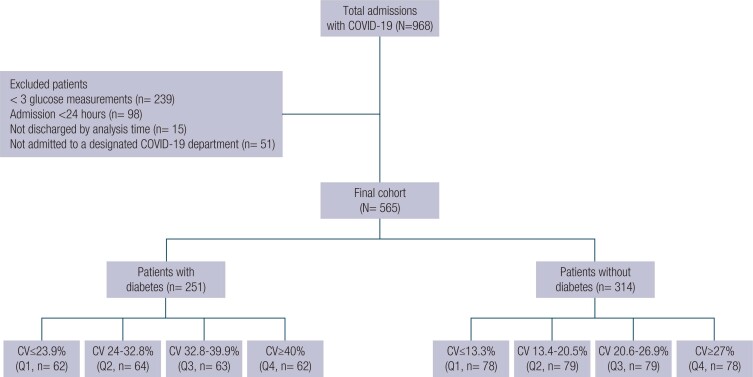
Flow diagram of study cohort.

CV: coefficient of variation; Q: quartile; COVID-19: coronavirus disease 19.

A total of 968 patients with COVID-19 were admitted to our medical center during the study period. After we excluded patients with fewer than three glucose measurements (239 patients), patients admitted for less than 24 hours (98 patients), patients still hospitalized during data extraction (15 patients), and those admitted in departments not designated for COVID-19 (51 patients), the final cohort included 565 patients (Figure 1). The mean age was 67.71 ± 15.45 years, and 62.3% were male. Of the entire cohort, 44.4% had DM. Those patients were older and had a higher prevalence of cardiovascular diseases and more severe COVID-19 on admission (Table 1). Of the patients with DM, 83.2% were treated with insulin during hospitalization.

**Table 1:** Characteristics of the study cohort

	Patients without diabetes (n = 314)	Patients with diabetes (n = 251)	P-value
Age, mean ± SD	65.1 ± 16.68	71 ± 13.08	<0.001
Male, n (%)	197 (62.74)	155 (61.75)	0.81
Body mass index, mean ± SD	28.9 ± 8.6	29.7 ± 10.09	0.35
**Comorbidities**			
Congestive heart failure, n (%)	11 (3.5)	32 (12.75)	<0.001
Hypertension, n (%)	90 (28.66)	147 (58.57)	<0.001
Obesity, n (%)	17 (5.41)	38 (15.14)	<0.001
Ischemic heart disease, n (%)	23 (7.32)	52 (20.72)	<0.001
Peripheral vascular disease, n (%)	3 (0.96)	7 (2.79)	0.10
Atrial fibrillation, n (%)	31 (9.87)	37 (14.74)	0.08
Dyslipidaemia, n (%)	22 (7.01)	40 (15.94)	0.001
Active malignancy, n (%)	21 (6.69)	20 (7.97)	0.56
COPD or asthma, n (%)	31 (9.87)	22 (8.76)	0.65
**Treatments during admission**			
Treated with antibiotics, n (%)	130 (41.4)	140 (55.78)	0.001
Treated with dexamethasone, n (%)	156 (49.68)	149 (59.36)	0.02
Treated with remdesivir, n (%)	62 (19.75)	58 (23.11)	0.33
Treated with anti-thrombotics, n (%)	187 (59.55)	154 (61.35)	0.66
Treated with insulin, n (%)	–	209 (83.2)	–
Treated with tocilizumab, n (%)	11 (3.50)	16 (6.37)	0.11
**Glucose data**			
Glucose coefficient of variation (%), mean ± SD	22.1 ± 15.75	33.2 ± 14.25	<0.001
**Plasma glucose value (mg/dL)**	119.79 ± 50.24	178.40 ± 82.34	<0.001
mean ± SD			
median (IQR)	110.3 (93.73-135)	160 (120-220)	
Last glucose value (mg/dL), mean ± SD	108.01 ± 33.98	162.93 ± 83.87	<0.001
**Number of glucose values per patient, median (IQR)**			
Quartile 1	4 (3-6)	13 (6.75-27.25)	<0.001
Quartile 2	7 (4-12)	29.5 (16.5-50.75)	
Quartile 3	7 (4-13)	32 (16-48)	
Quartile 4	43.5 (19.75-60.5)	8 (4.75-18.25)	
COVID-19 severity and laboratory data			
**COVID-19 severity at admission, n (%)**			
Mild	67 (21.34)	44 (17.53)	0.055
Moderate	27 (8.6)	33 (13.15)	
Severe	60 (19.11)	51 (20.32)	
Critical/Intubated	2 (0.64)	11 (4.38)	
Missing definition on admission	156 (49.7)	139 (55.4)	
Last WBC value (cells/mm^3^, mean ± SD)	8.01 ± 0.28	10.01 ± 0.47	<0.001
Last lymphocytes value (109/L, mean ± SD)	1.42 ± 0.07	1.36 ± 0.13	0.69
Last haemoglobin value (g/dL, mean ± SD)	12.13 ± 0.11	11.11 ± 0.13	<0.001
Last platelets value (109/L, mean ± SD)	278.08 ± 7.77	277.93 ± 10.05	0.99
Last CRP value (mg/L, mean ± SD)	6.29 ± 0.41	10.3 ± 0.73	<0.001
Last fibrinogen value (mg/dL, mean ± SD)	414.09 ± 9.04	426.37 ± 13.38	0.44
Last D-dimer value (ng/mL, mean ± SD)	3418.61 ± 689.04	5737.88 ± 1098.42	0.07
Last ferritin value (ng/mL, mean ± SD)	1008.56 ± 135.8	1294.59 ± 287.21	0.37
Last creatinine value (mg/dL, mean ± SD)	1.07 ± 0.06	1.68 ± 0.11	<0.001

COPD: chronic obstructive pulmonary disease; COVID-19: coronavirus disease 19; IQR: interquartile range; SD: standard deviation; WBC: white blood cells; CRP: C reactive protein. To convert mean glucose from mg/dL to mmol/liter, multiply by 0.0555.

### Glucose coefficient of variation

After stratifying the patients for DM diagnosis, we divided them into quartiles based on their glucose CV. In patients with DM, the median glucose CV was 32.8%, and in patients without DM, it was 20.5% (Figure 1). In the entire cohort, glucose CV ranged from 1% to 170%, but only 5 patients had a glucose CV higher than 70%. Patients in the upper glucose CV quartiles had more glucose measurements during admission (Table 1).

### Length of stay

In patients without DM, higher glucose CV was not associated with length of stay in the hospital in the unadjusted models (OR = 1.8, 95% CI [0.9,3.4] for Q4) or the adjusted model (OR = 1.7, 95% CI [0.7,4.2] for Q4 in model 3). However, in patients with DM, higher glucose CV was associated with longer hospitalization in the unadjusted model (OR = 2.7, 95% CI [1.3,5.6] for Q4) and when adjusted for age, sex, and comorbidities (OR = 3.1, 95% CI [1.3,7.3] for Q4). When we added adjustment for laboratory markers to the analysis, this association was no longer statistically significant (OR = 1.3, 95% CI [0.4,4.5] for Q4) (Table 2).

**Table 2:** Mortality and length of stay according to glucose coefficient of variation by quartiles in patients with and without diabetes mellitus.

			Patients Without Diabetes mellitus (n=314)				Patients With diabetes mellitus (n=251)			
			Q1	Q2	Q3	Q4	Q1	Q2	Q3	Q4
Length of stay	Median (IQR)		6 (3.75-9.25)	6 (4-11)	7 (3-10)	7.5 (5-13.25)	6 (2-10)	10 (5.25-16.75)	10 (6-15)	11.5 (6-19.25)
	OR (95% CI) [Length of stay >7 days]	Model 1^a^	–	1 (0.5 to 2)	1.2 (0.6 to 2.3)	1.8 (0.9 to 3.4)	–	2.2 (1.1 to 4.4)	2.4 (1.2 to 4.9)	2.7 (1.3 to 5.6)
		Model 2 b	–	1 (0.5 to 2.1)	1.2 (0.6 to 2.4)	1.2 (0.6 to 2.5)	–	1.8 (0.8 to 4.1)	2.5 (1.1 to 6)	3.1 (1.3 to 7.3)
		Model 3 c	–	0.8 (0.4 to 1.9)	0.7 (0.3 to 1.7)	1.7 (0.7 to 4.2)	–	1.8 (0.6 to 6.1)	1.2 (0.3 to 4.5)	1.3 (0.4 to 4.5)
	HR (95% CI)	Model 4 d	–	1.0 (0.7 to 1.5)	1.2 (0.8 to 1.9)	1.0 (0.6 to 1.5)	–	0.5 (0.3 to 0.9)	0.6 (0.3 to 1.1)	0.6 (0.3 to 1.0)
In-hospital mortality	No, (%)		4 (5.13)	6 (7.59)	10 (12.66)	18 (23.08)	11 (17.74)	13 (20.31)	27 (42.86)	29 (46.77)
	OR (95% CI)	Model 1 a	–	1.5 (0.4 to 5.6)	2.7 (0.8 to 8.9)	5.5 (1.8 to 17.3)	–	1.2 (0.5 to 2.9)	3.5 (1.5 to 7.9)	4.1 (1.8 to 9.3)
		Model 2 b	–	1.5 (0.3 to 7)	1.8 (0.4 to 8.3)	3.9 (1 to 16.1)	–	2.1 (0.7 to 6.9)	6.3 (2 to 19.2)	5.6 (1.9 to 17)
		Model 3 c	–	1.1 (0.1 to 10.2)	1.2 (0.1 to 11.4)	5.4 (0.7 to 39.3)	–	0.3 (0 to 2.1)	1.4 (0.2 to 9.4)	0.5 (0.1 to 3.4)
	HR (95% CI)	Model 4 d	–	0.0 (0.0 to 0.7)	17.0 (0.3 to 919.8)	0.4 (0.0 to 12.3)	–	0.8 (0.2 to 3.5)	0.7 (0.2 to 2.5)	0.6 (0.2 to 2.1)

a Model 1: Comparison with first quartile, unadjusted model.

b Model 2: Comparison with first quartile, adjusted for age, sex, body mass index, hypertension, dyslipidaemia, and atrial fibrillation.

c Model 3: Comparison with first quartile, adjusted for age, sex, body mass index, hypertension, dyslipidaemia, atrial fibrillation, last value of WBC, last value of d-dimer, last value of ferritin, and last value of CRP.

d Model 4: Same as model 3 but conducted with Cox multivariable analysis instead of logistic regression.

Q – quartile. OR – odds ratio, HR – hazard ratio, CI – confidence interval, IQR – interquartile range, WBC – white blood cells, CRP – C-reactive protein.

### In-hospital mortality

In patients without DM, higher glucose CV was associated with higher rates of in-hospital mortality in the unadjusted model (OR = 5.5, 95% CI [1.8,17.3] for Q4) and when adjusted to age, sex, and comorbidities (OR = 3.9, 95% CI [1,16.1] for Q4). When we added adjustment for laboratory markers to the analysis, this association was no longer statistically significant (OR = 5.4, 95% CI [0.7,39.3] for Q4). In patients with DM, the results were similar – the unadjusted model showed a significant association with in-hospital mortality (OR = 4.1, 95% CI [1.8,9.3] for Q4), but after adjustment for comorbidities and laboratory tests, the association was no longer statistically significant (OR = 0.5, 95% CI [0.1,3.4] for Q4 in model 3). Using Cox regression instead of logistic regression yielded similar results. The overall mortality rate was 12.1% among patients without DM and 31.9% among patients with DM (Table 2).

**Figure 2:**
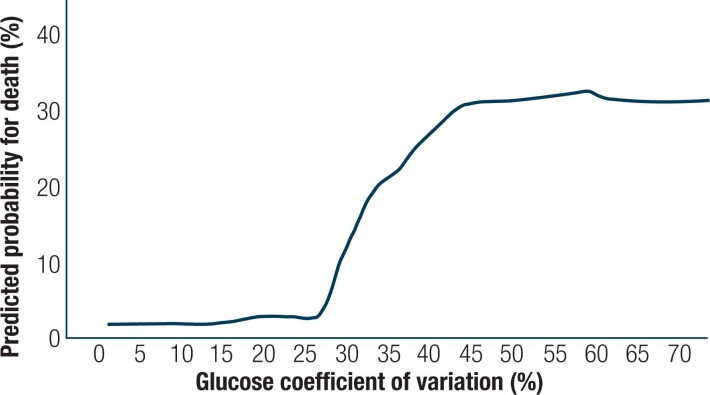
Predicted in-hospital mortality according to logistic regression model by glucose coefficient of variation in all patients presented by locally weighted scatterplot-smoothing (LOWESS) curve.

Figure 2 shows the results of a regression model that predicts in-hospital mortality using variables such as age, sex, comorbidities, and laboratory test results. The area under the curve for this model is 92.2%. Glucose CV was not a part of the model, and a LOWESS curve shows that when we assessed the entire cohort, glucose CV higher than 25% increased the probability of in-hospital mortality substantially.

## DISCUSSION

In this retrospective cohort study of hospitalized patients with COVID-19 with and without DM, higher glucose CV was associated with increased in-hospital mortality and length of stay, but this association disappeared when the adjustment included laboratory results data.

The glucose CV values were substantially higher in patients with and without DM than in cohorts of patients hospitalized in different wards in our institution (9,10,13). This finding is not surprising because a significant proportion of patients hospitalized with COVID-19 harbored significant inflammation (as the laboratory data in our cohort showed). In such conditions, catecholamine surges and endogenous cortisol production may increase glucose CV and be associated with worse outcomes. In addition, the universal use of dexamethasone as part of the standard protocol for the treatment of moderate to severe COVID-19 patients probably contributed significantly to glucose CV, as it increased post-prandial glucose levels (6,29). Of note, the rate of tocilizumab use in our cohort was low and hyperglycemia had been shown to have a negative effect on tocilizumab therapy in COVID-19 patients (30).

Hyperglycemia on admission, regardless of diabetes, was shown as a risk factor for progression to severe disease and mortality (31). Studies have shown that higher glucose CV was associated with higher rates of mortality and length of hospital stay. Previous studies were adjusted for age, sex, comorbidities, and hypoglycemia but not for any score of illness severity (9–11,13,32). Our results show that adjusting for various laboratory test results, which were shown to be a marker for COVID-19 severity of illness and mortality (33), eliminates the association between glucose CV and mortality. High glucose CV's negative effects are presumed to be fluctuations in oxidative stress, endothelial dysfunction, and increased inflammation (8,34). We suggest here that glucose CV is an additional marker of the body's inflammatory response to COVID-19 (35,36), and although it is not a significant predictor of mortality when adjusted for other acute inflammatory markers, it may serve as an easy-to-use marker of inflammatory and oxidative stress in hospitalized patients with COVID-19. As the COVID-19 pandemic continues to expand worldwide and methods of estimating disease severity are urgently required, glucose CV may serve as a readily available, cheap, and easy way to monitor method for this purpose. It is noteworthy that the threshold at which glucose CV was associated with mortality in our study was 25%, substantially lower than the 36% suggested in previous studies (37).

Our study has several limitations. First, its retrospective nature allowed us to determine only association. Second, patients with a history of abnormal glucose values have more glucose results, and the number of glucose measurements increases the glucose CV. Third, as previously noted, we cannot state whether increased glucose CV was a consequence or cause of the detrimental outcomes associated with high CV. Fourth, we did not have access to the community databases and could not determine glucose control prior to the admission. Despite these limitations, we assessed the prognostic value of glucose CV in hospitalized COVID-19 patients, and in the face of the pandemic, we believe this information will be found extremely useful for clinicians treating hospitalized COVID-19 patients.

In conclusion, among hospitalized COVID-19 patients with and without DM, increased in-hospital mortality and length of stay were associated with higher glucose CV, but this association disappears when we add acute inflammatory markers to the analysis. Future studies are needed to elucidate improved glucose CV's effect on in-hospital mortality and length of stay.
